# Neurobehavioral characteristics of mice with *SETD5* mutations as models of IDD23 and KBG syndromes

**DOI:** 10.3389/fgene.2022.1022339

**Published:** 2023-01-04

**Authors:** Tadashi Nakagawa, Satoko Hattori, Toru Hosoi, Keiko Nakayama

**Affiliations:** ^1^ Department of Clinical Pharmacology, Faculty of Pharmaceutical Sciences, Sanyo-Onoda City University, Sanyo-Onoda, Japan; ^2^ Division of Cell Proliferation, ART, Graduate School of Medicine, Tohoku University, Sendai, Miyagi, Japan; ^3^ Research Creation Support Center, Aichi Medical University, Nagakute, Aichi, Japan

**Keywords:** SETD5, IDD23, ANKRD11, KBG syndrome, mouse model

## Abstract

Genomic analysis has revealed that the genes for various chromatin regulators are mutated in many individuals with neurodevelopmental disorders (NDDs), emphasizing the important role of chromatin regulation in nervous system development and function. Chromatin regulation is mediated by writers, readers, and erasers of histone and DNA modifications, with such proteins being defined by specific domains. One of these domains is the SET domain, which is present in enzymes that catalyze histone methylation. Heterozygous loss-of-function mutations of the *SETD5* (SET domain containing 5) gene have been identified in individuals with an NDD designated IDD23 (intellectual developmental disorder, autosomal dominant 23). KBG syndrome (named after the initials of the last names of the first three families identified with the condition) is characterized by features that either overlap with or are distinct from those of IDD23 and was initially thought to be caused only by mutations in the *ANKRD11* (ankyrin repeat domain containing 11) gene. However, recent studies have identified *SETD5* mutations in some KBG syndrome patients without *ANKRD11* mutations. Here we summarize the neurobehavioral characterization of *Setd5* ^*+/−*^ mice performed by four independent research groups, compare IDD23 and KBG phenotypes, and address the utility and future development of mouse models for elucidation of the mechanisms underlying NDD pathogenesis, with a focus on SETD5 and its related proteins.

## Introduction

The regulation of gene expression relies on chromatin structure, which is itself dependent on DNA and histone modifications such as methylation of lysine residues in the NH_2_-terminal tails of histone proteins (Berger, 2007). Most histone lysine methyltransferases contain an evolutionarily conserved SET (Suppressor of variegation 3, Enhancer of Zeste, and Trithorax) domain that binds the *S*-adenosyl methionine (SAM) cofactor required for catalysis of methylation (Zhang and Ma, 2012; Herz et al., 2013; Husmann and Gozani, 2019). Given that it contains a SET domain, mammalian SETD5 might be expected to function as a histone methyltransferase. However, the SET domain of SETD5 differs at several key residues from those of active methyltransferases as well as contains a large loop not present in such enzymes, both of which features disrupt SAM binding (Mas-y-Mas et al., 2016), leaving the catalytic activity of SETD5 unclear. Whereas H3K36 (lysine-36 of histone H3) methylation (Sessa et al., 2019) and H3K9 methylation (Pinheiro et al., 2012) were reported to be mediated by the recombinant SET domain of SETD5 *in vitro*, other researchers were not able to detect such activity (Deliu et al., 2018; Wang et al., 2020). Methyltransferase activity has also not been detected for the SETD5 paralog MLL5 (Madan et al., 2009; Mas-y-Mas et al., 2016) or for SETD5 orthologs including yeast Set3p (Pijnappel et al., 2001) and *Drosophila* UpSET (Rincon-Arano et al., 2012), suggesting that, if SETD5 indeed possesses such catalytic activity, it was acquired during the process of evolution. Proteomics analyses by several research groups have consistently identified components of two complexes—the HDAC3 and PAF1 complexes—among proteins found to associate with SETD5 (Osipovich et al., 2016; Yu et al., 2017; Deliu et al., 2018; Nakagawa et al., 2020; Wang et al., 2020; Matsumura et al., 2021). The HDAC3 complex functions mainly as a transcriptional repressor by catalyzing deacetylation of histone proteins (Seto and Yoshida, 2014), whereas the PAF1 complex regulates transcription by modulating promoter-proximal pausing, transcriptional elongation and termination, and RNA 3′-end formation by RNA polymerase II (Branden Van Oss et al., 2017). Therefore, regardless of whether it possesses catalytic activity itself, SETD5 is implicated in chromatin regulation and is classified as an epigenetic regulator that acts through the HDAC3 and PAF1 complexes. The identification of genes regulated by SETD5 in association with HDAC3 and PAF1 complexes has provided insight into physiology and pathology related to cancer or to neurodevelopmental disorders (NDDs), in which up- or downregulation of SETD5 expression has been detected, respectively.

Intellectual developmental disorders (IDDs) constitute a spectrum of NDDs associated with intellectual disability (ID), and they impact physical, cognitive, or emotional functions of affected individuals. Genetic alterations of several autosomal dominant genes associated with IDDs have been identified and numbered, with MRD1 (“mental retardation”, autosomal dominant 1) referring to mutation or disruption of the gene *MBD5* and MRD2 to disruption of *DOCK8*. Since the abbreviation “MR” for “mental retardation” is being replaced with “ID” for intellectual disability, here we use “IDD” instead of “MRD” in this Mini Review. In most cases, each IDD number is assigned to a single gene or chromosomal location whose genetic alteration is responsible for the corresponding disorder. Of the 69 IDDs annotated in the OMIM (Online Mendelian Inheritance in Man) database, at least 36 (52%) are linked to genes whose products (proteins) are molecularly related to chromatin regulation and transcription (Table 1), underlining the key role of the epigenetic control of gene expression in nervous system development.

**TABLE 1:** Genes associated with MRD in the OMIM database. Given that the term “mental retardation (MR)” is being replaced in the literature with “intellectual disability (ID),” we propose that “MRD (“mental retardation,” autosomal dominant)” be replaced with “IDD (intellectual disability, autosomal dominant).” Chromatin regulators are defined as those related to transcription directly such as transcription factors or epigenetic regulators.

MRD (IDD) number	Autosomal dominant ID gene	Chromatin regulator	Type of mutation	Location
1	MBD5	Yes	deletion, duplication, substitution, translocation	2q23.1
2	DOCK8		deletion, translocation	9p24
3	CDH15		substitution, translocation	16q24.3
4	KIRREL3		substitution, translocation	11q24.2
5	SYNGAP1		deletion, substitution	6p21.32
6	GRIN2B		deletion, insertion, substitution, translocation	12p13.1
7	DYRK1A		deletion, substitution, translocation	21q22.13
8	GRIN1		duplication, substitution	9q34.3
9	KIF1A		substitution	2q37.3
10	CACNG2		substitution	22q12.3
11	EPB41L1		substitution	20q11.23
12	ARID1B	Yes	deletion, substitution	6q25.3
13	DYNC1H1		substitution	14q32.31
14	ARID1A	Yes	deletion, duplication, substitution	1p36.11
15	SMARCB1	Yes	deletion, substitution	22q11.23
16	SMARCA4	Yes	deletion, substitution	19p13.2
17	PACS1		substitution	11q13.1-q13.2
18	GATAD2B	Yes	deletion, insertion, substitution	1q21.3
19	CTNNB1	Yes	deletion, insertion, duplication, substitution	3p22.1
20	MEF2C	Yes	deletion, duplication, substitution	5q14.3
21	CTCF	Yes	duplication, substitution	16q22.1
22	ZBTB18	Yes	deletion, substitution	1q44
23	SETD5	Yes	deletion, duplication, substitution	3p25.3
24	DEAF1	Yes	deletion, deletion/insertion, substitution	11p15.5
25	AHDC1	Yes	deletion, substitution	1p36.11-p35.3
26	AUTS2	Yes	deletion, duplication, translocation, inversion	7q11.22
27	SOX11	Yes	substitution	2p25.2
28	ADNP	Yes	deletion, insertion, duplication, substitution	20q13.13
29	SETBP1	Yes	deletion, substitution	18q12.3
30	ZMYND11	Yes	deletion, duplication, substitution	10p15.3
31	PURA	Yes	deletion, substitution	5q31.3
32	KAT6A	Yes	deletion, duplication, substitution	8p11.21
33	DPP6		deletion, substitution	7q36.2
34	CERT1		substitution	5q13.3
35	PPP2R5D		substitution	6p21.1
36	PPP2R1A		substitution	19q13.41
37	POGZ	Yes	deletion, insertion, duplication, substitution	1q21.3
38	EEF1A2		substitution	20q13.33
39	MYT1L	Yes	deletion, duplication, substitution	2p25.3
40	CHAMP1	Yes	deletion, substitution	13q34
41	TBL1XR1	Yes	deletion, substitution	3q26.32
42	GNB1		deletion, substitution	1p36.33
43	HIVEP2	Yes	deletion, duplication, substitution	6q24.2
44	TRIO		deletion, duplication, substitution	5p15.2
45	CIC	Yes	deletion/insertion, duplication, substitution	19q13.2
46	KCNQ5		substitution	6q13
47	STAG1	Yes	deletion, duplication, substitution	3q22.3
48	RAC1		substitution	7p22.1
49	TRIP12	Yes	deletion, duplication, substitution	2q36.3
50	NAA15		deletion, deletion/insertion, substitution	4q31.1
51	KMT5B	Yes	deletion, substitution	11q13.2
52	ASH1L	Yes	deletion/insertion, duplication, substitution	1q22
53	CAMK2A		substitution	5q32
54	CAMK2B		substitution	7p13
55	NUS1		deletion, duplication, substitution	6q22.1
56	CLTC		deletion, duplication, substitution	17q23.1
57	TLK2	Yes	substitution, translocation	17q23.2
58	SET	Yes	deletion, duplication, substitution	9q34.11
59	CAMK2G		substitution	10q22.2
60	AP2M1		substitution	3q27.1
61	MED13	Yes	deletion, substitution	17q23.2
62	DLG4		deletion, duplication, substitution	17p13.1
63	TRIO		substitution	5p15.2
64	ZNF292	Yes	deletion, duplication, substitution	6q14.3
65	KDM4B	Yes	deletion, duplication, substitution	19p13.3
66	ATP2B1		substitution	12q21.33
67	GRIA1		substitution	5q33.2
68	KMT2B	Yes	duplication, substitution	19q13.12
69	LMAN2L		deletion	2q11.2

IDD23 is defined as being caused by a heterozygous mutation of *SETD5* on chromosome 3p25 in humans, with the corresponding mouse gene being located at chromosome 6 cytoband E3 (Fernandes et al., 2018). Individuals with a 3p25 deletion manifest an array of symptoms including ID, autistic features, epilepsy, eye problems, facial dysmorphism, and congenital heart defects (Shuib et al., 2009; Gunnarsson and Bruun, 2010; Peltekova et al., 2012; Kellogg et al., 2013; Pinto et al., 2014; Pires et al., 2020) (Table 2; Supplementary Table S1). The deletions overlap with a portion, or the entire region, of *SETD5*, resulting in the loss of gene function. Missense mutations, frameshift mutations, or non-sense mutations of *SETD5* have also been detected in individuals who manifest phenotypes similar to those associated with 3p25 deletion (Rauch et al., 2012; Grozeva et al., 2014; Kuechler et al., 2015; Szczałuba et al., 2016; Green et al., 2017; Stur et al., 2017; Powis et al., 2018; Fang et al., 2019; Pan et al., 2020; Pinard et al., 2020; Anderson et al., 2021) (Supplementary Figure S1). Although the link between the types of mutations in the *SETD5* gene and particular phenotypes such as ID is not obvious (Supplementary Table S1), those patients’ data indicated that *SETD5* is responsible, at least in part, for the 3p25 deletion syndrome. The causal role of SETD5 insufficiency due to heterozygous loss-of-function mutations in disease pathogenesis has been investigated with mouse models. Here we summarize the insight provided by these models and examine the extent to which the mice recapitulate human disease phenotypes. We will also address KBG syndrome, which was thought to be caused specifically by *ANKRD11* (ankyrin repeat domain containing 11) gene mutations, but for which a role for *SETD5* was recently suggested (Crippa et al., 2020; Pascolini et al., 2022).

**TABLE 2:** Clinical characteristics of SETD5 haploinsufficiency in humans and mice. The references for human data are found in the Supplementary Table. Mouse data are from Deliu et al. (2018), Moore et al. (2019), Sessa et al. (2019), Nakagawa et al. (2020), and Cheung et al. (2021) and each study is represented as D, M, S, N, and C, respectively, at the right-most column. N.D., not determined.

Human		Mouse		
Clinical feature	Penetrance	Phenotype	Penetrance	Reference
Intellectual disability	90%	Abnormal fear memory, impaired working memory	N.D.	D, M, S, N
Autistic features	50%	Impaired social behavior, repetitive nose poking	N.D.	D, M, S, N
Epilepsy	25%	Abnormal electroencephalogram	0%	M
Eye problems	65%	Corectopia, mydriasis, and microphthalmia	15%	D
Facial dysmorphism	100%	Craniofacial abnormalities, tooth displacement	N.D.	D
Congenital heart defects	40%	Defects in heart development	75%	C
Motor impairment	90%	Abnormal recurrent paw slipping	N.D.	S

### Mouse models of SETD5 insufficiency

Mice with an insertion of the coding sequence for green fluorescent protein (GFP) upstream of the *Setd5* translation initiation codon have been generated and analyzed (Osipovich et al., 2016). These mice express GFP in place of SETD5, and the homozygous disruption of *Setd5* was found to result in death at around embryonic day 10 that was likely due to a cardiovascular abnormality. The SETD5 null embryos thus manifested an underdeveloped heart, widespread hemorrhage, and a disorganized capillary plexus with fewer blood cells in the yolk sac, which nurtures the embryo proper. Consistent with these findings, the differentiation of SETD5-deficient embryonic stem cells into cardiomyocytes was impaired in association with dysregulation of the expression of genes related to such differentiation.

Four research groups subsequently and independently characterized the neurobehavioral phenotypes of *Setd5* ^*+/−*^ mice, with one group studying the mice lacking all exons of *Setd5* generated by Osipovich et al. (Moore et al., 2019) and the other groups studying mice established by the Wellcome Trust Sanger Institute Mouse Genetics Project (Pettitt et al., 2009; Skarnes et al., 2011; Bradley et al., 2012; White et al., 2013). This latter mouse line, *Setd5* ^+/tm1a (EUCOMM)Wtsi^, contains a transcription stop sequence in intron 2 that can be excised with flippase (Flp) as well as loxP sites flanking exons 3 to 6 that allow this genomic region to be excised with Cre recombinase [see Supplementary Figure S1 in Nakagawa et al. (2020)]. The induced expression of Cre in specific cell types can thus result in the conditional knockout of *Setd5* in these cell types. We (Nakagawa et al., 2020) and Sessa et al. (2019) analyzed these mice lacking exons 3 to 23, without the use of Cre and Flp driver mice, whereas Deliu et al. (2018) generated mice lacking exons 3 to 6 by crossing *Setd5* ^+/tm1a (EUCOMM)Wtsi^ mice with Flp-expressing mice and mating the resultant offspring with Cre transgenic mice that express Cre throughout the body (Supplementary Figure S1). We will now summarize the major NDD phenotypes of these various *Setd5* ^*+/−*^ mice.

### Cognitive function

ID is characterized by impaired cognitive function and adaptive behavior (The Diagnostic and Statistical Manual of Mental Disorders, Fifth Edition). The cognitive ability of *Setd5* heterozygous mutant mice was evaluated by subjecting the animals to several learning and memory tests, including fear-conditioning and maze tests. In our analysis (Nakagawa et al., 2020), the mice lacking exons 3 to 23 of *Setd5* manifested reduced freezing behavior induced by a pretrained sound stimulus, indicative of an impairment of cued fear memory. Although we detected no significant defect in working memory performance in the T-maze test, mice with the same targeted deletion of *Setd5* showed impaired performance in the working memory task of the eight-arm radial maze test (Sessa et al., 2019). Consistent with our results (Nakagawa et al., 2020), the *Setd5* ^*+/−*^ mice studied by Moore et al. (2019) showed a significantly increased latency in the Barnes maze test compared with wild-type animals. In contrast, Deliu et al. (2018) found that the *Setd5* mutant mice lacking exons 3 to 6 manifested increased memory retention as assessed by the contextual fear memory test and the ability to discriminate between old and novel locations. A large deletion of *Setd5*, including the region encoding the COOH-terminus of the protein, thus resulted in cognitive impairment, whereas the opposite phenotype was observed in mice with a smaller deletion. In addition, the mutant mice lacking exons 3 to 6 of *Setd5* showed repetitive behavior and deficits in adaptive behavior with the IntelliCage system (Deliu et al., 2018), which may have influenced memory performance.

### Social interaction and repetitive behavior

Autism spectrum disorder (ASD) is characterized by impairment of social interaction and communication and the presence of repetitive behaviors (The Diagnostic and Statistical Manual of Mental Disorders, Fifth Edition). Social interaction was investigated in mice with heterozygous deletion of *Setd5* with the use of several types of social behavior tests, including a three-chamber social approach test and ultrasonic vocalization (USV) recording. Whereas we (Nakagawa et al., 2020) and Deliu et al. (2018) did not detect any significant defects in the three-chamber social approach test, the other two groups found that *Setd5* ^*+/−*^ mice lacked the normal preference for novel over familiar mice (Moore et al., 2019; Sessa et al., 2019). In the social interaction test performed with two mice of the same genotype, we found that the mean duration per contact was significantly increased and the total duration of contacts tended to be increased for *Setd5* ^*+/−*^ mice compared with wild-type littermates (Nakagawa et al., 2020). However, Sessa et al. (2019) observed a reduced total duration of contacts for their *Setd5* mutant animals.

The communicative function of USV has been demonstrated in young mouse pups (Scattoni et al., 2009). We assessed social communication in *Setd5* ^*+/−*^ mice by measurement of USV at postnatal day 6, and found that the mutant mice emitted fewer such vocalizations than did wild-type mice (Nakagawa et al., 2020). *Setd5* ^*+/−*^ mouse pups also showed a delayed ontogenetic profile of USV (Deliu et al., 2018). In the nest-building task, the mutant mice also failed to build proper nests (Deliu et al., 2018; Moore et al., 2019), and they were subordinate to wild-type mice in the tube test (Sessa et al., 2019).

The mutant mice were assessed for repetitive behavior with the marble-burying and open-field tests, in which they performed similarly to control mice, whereas the mutant animals showed nose-poke repetitions in a learning task performed with IntelliCage (Deliu et al., 2018). Together, these data indicated that, regardless of the size of the gene deletion, SETD5 haploinsufficiency appeared to give rise to social behavioral abnormalities reminiscent of autistic-like behavior.

### Other features

Epilepsy is a frequent complication of individuals with *SETD5* mutations. However, *Setd5* mutant mice did not show spontaneous seizures or a reduction in the threshold for drug-induced epilepsy (Moore et al., 2019). Individuals with *SETD5* mutations also experience motor impairment or delay, but motor coordination in the rotarod test was also normal in *Setd5* ^*+/−*^ mice (Moore et al., 2019; Sessa et al., 2019; Nakagawa et al., 2020)—although the mutant mice showed a severe impairment in hindlimb clasping and abnormal paw slipping in the beam-walking test (Sessa et al., 2019). Many, but not all, *Setd5* ^*+/−*^ mice manifested tooth displacement, eye problems, and craniofacial abnormalities (Deliu et al., 2018), all of which are characteristics of patients harboring *SETD5* mutations. Recently, more than half of *Setd5* ^*+/−*^ mice were also found to have congenital heart defects (Cheung et al., 2021), which are also observed in human patients. Together, these results indicate that SETD5 haploinsufficiency affects developmental processes in a variety of organs of both humans and mice.

### KBG syndrome

Heterozygous mutations of *ANKRD11* have been identified in individuals with KBG syndrome, which is named after the initials of the last names of the first three families identified with the condition and is characterized by short stature, macrodontia of permanent upper central incisors, facial dysmorphism, learning difficulties, and neurobehavioral problems; it is also associated less frequently with seizures, cardiac abnormalities, hearing loss, feeding difficulties, and autistic features (Low et al., 2016). A search for novel pathogenic variants in three patients with suspected KBG syndrome who did not harbor *ANKRD11* mutations resulted in the identification of an exon 1–17 deletion and frameshift mutations in *SETD5* (Crippa et al., 2020). More recently, a girl with KBG syndrome features but without an *ANKRD11* mutation was found to harbor a non-sense mutation in *SETD5* (Pascolini et al., 2022). Of note, we (Nakagawa et al., 2020) and Deliu et al. (2018) detected ANKRD11 in SETD5 immunoprecipitates prepared from neuronal cells, indicating that ANKRD11 binds to SETD5 and may function as a transcriptional repressor in the same complex. This notion is supported by the observation that ANKRD11 also binds to HDAC3 (Zhang et al., 2004), which also interacts with SETD5. The proliferation of cultured cortical precursor cells from Yoda mice, which harbor a missense mutation of *Ankrd11* similar to a mutation identified in a KBG syndrome patient, was found to be impaired, and the defect was rescued by ectopic overexpression of HDAC3 (Gallagher et al., 2015). Combined with our finding that neural stem cells derived from *Setd5* mutant mice also showed reduced proliferation (Nakagawa et al., 2020), these data indicate that SETD5 functions together with ANKRD11 in the HDAC3 complex, and probably possibly also the PAF1 complex, to regulate gene expression that is necessary for neural development. In the three-chamber social approach test, Yoda mice showed impaired sociability and preference for novel over familiar mice as *Setd5* ^*+/−*^ mice did (Gallagher et al., 2015). Furthermore, these mice exhibited more self-grooming behavior than their wild-type littermates did (Gallagher et al., 2015), indicating that *Ankrd11* mutation causes ASD-like social deficit in mice.

## Discussion

We have here surveyed studies that have analyzed mouse models related to IDD23 and KBG syndrome, namely *Setd5* ^*+/−*^ and *Ankrd11* ^Yoda^ mice. Similar to human patients, these mice manifest a variety of phenotypes with less than 100% penetrance, implicating environmental factors as determinants of such phenotypes. The intestinal environment is of particular interest in this regard, given that the gut-brain axis is proposed to play a key role in ASD pathogenesis (Vuong et al., 2017; Cryan et al., 2019). It will be important to identify environmental factors responsible for this phenotypic heterogeneity in order to increase our understanding of these NDDs.

Several issues remain to be clarified with regard to the pathology associated with *SETD5* mutation. The molecular relation between SETD5 and its target genes thus remains undefined. Given that SETD5 does not contain a DNA binding domain, it must rely on other proteins for its recruitment to target loci. Moreover, the function of the SET domain of SETD5 is still not clear. Of interest, a comparison of the sequence similarity between human and chimpanzee SET domains revealed more non-synonymous substitutions in SETD5 than in any other SET domain–containing protein, indicative of rapid evolution of SETD5 in humans (Zhang and Ma, 2012). A human-specific function of the SET domain of SETD5 may exist whose disruption gives rise to IDD23 pathogenesis.

Whether there is a link between NDDs and tumorigenesis also warrants further investigation. Whereas an increase in SETD5 expression or activity may ameliorate the symptoms of IDD23, the effect of such an increase on tumorigenesis will need to be determined, given that SETD5 is upregulated in most cancer types (Ding et al., 2019; Yu et al., 2019; Zhang et al., 2019; Piao et al., 2020; Wang et al., 2020; Chen et al., 2021; Yang et al., 2021; Jiang, et al., 2022; Park et al., 2022; Yang et al., 2022).

IDD23 and KBG syndrome are both mendelian disorders of the epigenetic machinery (MDEMs), an emerging concept that emphasizes the importance of epigenetic regulators in disorders such as NDDs and growth defects with prominent sensitivity to haploinsufficiency as a key pathogenic feature (Fahrner and Bjornsson, 2019). Further studies of mice bearing various mutations of *Setd5* as well as other MDEM mouse models, with or without infliction of environmental insults, should help to characterize how the nervous system maintains homeostasis in order to execute its functions during development. For example, single-cell transcriptomic analyses of brain samples taken from *Setd5* ^*+/−*^ and *Ankrd11* ^Yoda^ mice or behavioral and biochemical examination of mice with conditional knockout of *Setd5* or *Ankrd11* would be helpful to determine which cell types require SETD5 for preventing the pathogenesis of NDD/KBG syndrome.

Finally, the abbreviation “MR” for “mental retardation” is being replaced with “ID” for intellectual disability, and we therefore propose that “MRD” be replaced with “IDD” in this numbering system.

## References

[B1] Anderson E. Lam Z. Arundel P. Parker M. Balasubramanian M. (2021). Expanding the phenotype of SETD5-related disorder and presenting a novel association with bone fragility. Clin. Genet. 100, 352–354. 10.1111/cge.14014 34169511

[B2] Berger S. L. (2007). The complex language of chromatin regulation during transcription. Nature 447, 407–412. 10.1038/nature05915 17522673

[B3] Bradley A. Anastassiadis K. Ayadi A. Battey J. F. Bell C. Birling M.-C. (2012). The mammalian gene function resource: The international knockout mouse consortium. Mamm. Genome 23, 580–586. 10.1007/s00335-012-9422-2 22968824 PMC3463800

[B4] Branden Van Oss S. Cucinotta C. E. Arndt K. M. (2017). Emerging insights into the roles of the Paf1 complex in gene regulation. Trends biochem. Sci. 42, 788–798. 10.1016/j.tibs.2017.08.003 28870425 PMC5658044

[B5] Chen Q. Sun Z. Li J. Zhang D. Guo B. Zhang T. (2021). SET domain-containing protein 5 enhances the cell stemness of non-small cell lung cancer via the PI3K/Akt/mTOR pathway. J. Environ. Pathol. Toxicol. Oncol. 40, 55–63. 10.1615/JEnvironPatholToxicolOncol.2021036991 33822517

[B6] Cheung M. Y.-Q. Roberts C. Scambler P. Stathopoulou A. (2021). Setd5 is required in cardiopharyngeal mesoderm for heart development and its haploinsufficiency is associated with outflow tract defects in mouse. Genesis 59, e23421. 10.1002/dvg.23421 34050709 PMC8564859

[B7] Crippa M. Bestetti I. Maitz S. Weiss K. Spano A. Masciadri M. (2020). SETD5 gene haploinsufficiency in three patients with suspected KBG syndrome. Front. Neurol. 11, 631. 10.3389/fneur.2020.00631 32793091 PMC7393934

[B8] Cryan J. F. O’riordan K. J. Cowan C. S. M. Sandhu K. V. Bastiaanssen T. F. S. Boehme M. (2019). The microbiota-gut-brain axis. Physiol. Rev. 99, 1877–2013. 10.1152/physrev.00018.2018 31460832

[B9] Deliu E. Arecco N. Morandell J. Dotter C. P. Contreras X. Girardot C. (2018). Haploinsufficiency of the intellectual disability gene SETD5 disturbs developmental gene expression and cognition. Nat. Neurosci. 21, 1717–1727. 10.1038/s41593-018-0266-2 30455454

[B10] Ding B. Yan L. Zhang Y. Wang Z. Zhang Y. Xia D. (2019). Analysis of the role of mutations in the KMT2D histone lysine methyltransferase in bladder cancer. FEBS Open Biol. 9, 693–706. 10.1002/2211-5463.12600 PMC6443872 30984543

[B11] Fahrner J. A. Bjornsson H. T. (2019). Mendelian disorders of the epigenetic machinery: Postnatal malleability and therapeutic prospects. Hum. Mol. Genet. 28, R254-R264–R264. 10.1093/hmg/ddz174 31595951 PMC6872430

[B12] Fang Y.-L. Zhang R.-P. Wang Y.-Z. Cao L.-R. Zhang Y.-Q. Cai C.-Q. (2019). A novel mutation in a common pathogenic gene (SETD5) associated with intellectual disability: A case report. Exp. Ther. Med. 18, 3737–3740. 10.3892/etm.2019.8059 31656537 PMC6812316

[B13] Fernandes I. R. Cruz A. C. P. Ferrasa A. Phan D. Herai R. H. Muotri A. R. (2018). Genetic variations on SETD5 underlying autistic conditions. Dev. Neurobiol. 78, 500–518. 10.1002/dneu.22584 29484850

[B14] Gallagher D. Voronova A. Zander M. A. Cancino G. I. Bramall A. Krause M. P. (2015). Ankrd11 is a chromatin regulator involved in autism that is essential for neural development. Dev. Cell. 32, 31–42. 10.1016/j.devcel.2014.11.031 25556659

[B15] Green C. Willoughby J. Balasubramanian M. (2017). De novo SETD5 loss-of-function variant as a cause for intellectual disability in a 10-year old boy with an aberrant blind ending bronchus. Am. J. Med. Genet. A 173, 3165–3171. 10.1002/ajmg.a.38461 28905509

[B16] Grozeva D. Carss K. Spasic-Boskovic O. Parker M. J. Archer H. Firth H. V. (2014). De novo loss-of-function mutations in SETD5, encoding a methyltransferase in a 3p25 microdeletion syndrome critical region, cause intellectual disability. Am. J. Hum. Genet. 94, 618–624. 10.1016/j.ajhg.2014.03.006 24680889 PMC3980521

[B17] Gunnarsson C. Bruun C. F. (2010). Molecular characterization and clinical features of a patient with an interstitial deletion of 3p25.3-p26.1. Am. J. Med. Genet. A 152A, 3110–3114. 10.1002/ajmg.a.33353 21082655

[B18] Herz H.-M. Garruss A. Shilatifard A. (2013). SET for life: Biochemical activities and biological functions of SET domain-containing proteins. Trends biochem. Sci. 38, 621–639. 10.1016/j.tibs.2013.09.004 24148750 PMC3941473

[B19] Husmann D. Gozani O. (2019). Histone lysine methyltransferases in biology and disease. Nat. Struct. Mol. Biol. 26, 880–889. 10.1038/s41594-019-0298-7 31582846 PMC6951022

[B20] Jiang Z. Zhao J. Zho H. Cai K. (2022). CircRNA PTPRM promotes non-small cell lung cancer progression by modulating the miR-139-5p/SETD5 Axis. Technol. Cancer Res. Treat. 21, 15330338221090090. 10.1177/15330338221090090 35491723 PMC9066640

[B21] Kellogg G. Sum J. Wallerstein R. (2013). Deletion of 3p25.3 in a patient with intellectual disability and dysmorphic features with further definition of a critical region. Am. J. Med. Genet. A 161, 1405–1408. 10.1002/ajmg.a.35876 23613140

[B22] Kuechler A. Zink A. M. Wieland T. Lüdecke H.-J. Cremer K. Salviati L. (2015). Loss-of-function variants of SETD5 cause intellectual disability and the core phenotype of microdeletion 3p25.3 syndrome. Eur. J. Hum. Genet. 23, 753–760. 10.1038/ejhg.2014.165 25138099 PMC4795044

[B23] Low K. Ashraf T. Canham N. Clayton-Smith J. Deshpande C. Donaldson A. (2016). Clinical and genetic aspects of KBG syndrome. Am. J. Med. Genet. A 170, 2835–2846. 10.1002/ajmg.a.37842 27667800 PMC5435101

[B24] Madan V. Madan B. Brykczynska U. Zilbermann F. Hogeveen K. Döhner K. (2009). Impaired function of primitive hematopoietic cells in mice lacking the Mixed-Lineage-Leukemia homolog MLL5. Blood 113, 1444–1454. 10.1182/blood-2008-02-142638 18952892

[B25] Mas-y-Mas S. Barbon M. Teyssier C. Déméné H. Carvalho J. E. Bird L. E. (2016). The human mixed lineage leukemia 5 (MLL5), a sequentially and structurally divergent SET domain-containing protein with no intrinsic catalytic activity. PLoS One 11, e0165139. 10.1371/journal.pone.0165139 27812132 PMC5094779

[B26] Matsumura Y. Ito R. Yajima A. Yamaguchi R. Tanaka T. Kawamura T. (2021). Spatiotemporal dynamics of SETD5-containing NCoR-HDAC3 complex determines enhancer activation for adipogenesis. Nat. Commun. 12, 7045. 10.1038/s41467-021-27321-5 34857762 PMC8639990

[B27] Moore S. M. Seidman J. S. Ellegood J. Gao R. Savchenko A. Troutman T. D. (2019). Setd5 haploinsufficiency alters neuronal network connectivity and leads to autistic-like behaviors in mice. Transl. Psychiatry 9, 24. 10.1038/s41398-018-0344-y 30655503 PMC6336863

[B28] Nakagawa T. Hattori S. Nobuta S. Kimura R. Nakagawa M. Matsumoto M. (2020). The autism-related protein SETD5 controls neural cell proliferation through epigenetic regulation of rDNA expression. iScience 23, 101030. 10.1016/j.isci.2020.101030 32299058 PMC7160574

[B29] Osipovich A. B. Gangula R. Vianna P. G. Magnuson M. A. (2016). Setd5 is essential for mammalian development and the co-transcriptional regulation of histone acetylation. Development 143, 4595–4607. 10.1242/dev.141465 27864380 PMC5201031

[B30] Pan M. Liu Y. N. Xu L. L. Li D. Z. (2020). First-trimester cystic hygroma and neurodevelopmental disorders: The association to remember. Taiwan J. Obstet. Gynecol. 59, 960–962. 10.1016/j.tjog.2020.09.029 33218422

[B31] Park M. Moon B. Kim J-H. Park S-J. Kim S-K. Park K. (2022). Downregulation of SETD5 suppresses the tumorigenicity of hepatocellular carcinoma cells. Mol. Cells 45, 550–563. 10.14348/molcells.2022.0009 35950456 PMC9385566

[B32] Pascolini G. Gnazzo M. Novelli A. Grammatico P. (2022). Clinical refinement of the SETD5-associated phenotype in a child displaying novel features and KBG syndrome-like appearance. Am. J. Med. Genet. A 188, 1623–1625. 10.1002/ajmg.a.62679 35132768

[B33] Peltekova I. T. Macdonald A. Armour C. M. (2012). Microdeletion on 3p25 in a patient with features of 3p deletion syndrome. Am. J. Med. Genet. A 158, 2583–2586. 10.1002/ajmg.a.35559 22903836

[B34] Pettitt S. J. Liang Q. Rairdan X. Y. Moran J. L. Prosser H. M. Beier D. R. (2009). Agouti C57BL/6N embryonic stem cells for mouse genetic resources. Nat. Methods 6, 493–495. 10.1038/nmeth.1342 19525957 PMC3555078

[B35] Piao L. Li H. Feng Y. Yang Z. Kim S. Xuan Y. (2020). SET domain-containing 5 is a potential prognostic biomarker that promotes esophageal squamous cell carcinoma stemness. Exp. Cell. Res. 389, 111861. 10.1016/j.yexcr.2020.111861 31981592

[B36] Pijnappel W. W. Schaft D. Roguev A. Shevchenko A. Tekotte H. Wilm M. (2001). The *S. cerevisiae* SET3 complex includes two histone deacetylases, Hos2 and Hst1, and is a meiotic-specific repressor of the sporulation gene program. Genes. Dev. 15, 2991–3004. 10.1101/gad.207401 11711434 PMC312828

[B37] Pinard A. Guey S. Guo D. Cecchi A. C. Kharas N. Wallace S. (2020). The pleiotropy associated with de novo variants in CHD4, CNOT3, and SETD5 extends to moyamoya angiopathy. Genet. Med. 22, 427–431. 10.1038/s41436-019-0639-2 31474762 PMC7673309

[B38] Pinheiro I. Margueron R. Shukeir N. Eisold M. Fritzsch C. Richter F. M. (2012). Prdm3 and Prdm16 are H3K9me1 methyltransferases required for mammalian heterochromatin integrity. Cell. 150, 948–960. 10.1016/j.cell.2012.06.048 22939622

[B39] Pinto D. Delaby E. Merico D. Farbosa M. Merikangas A. Klei L. (2014). Convergence of genes and cellular pathways dysregulated in autism spectrum disorders. Am. J. Hum. Genet. 94, 677–694. 10.1016/j.ajhg.2014.03.018 24768552 PMC4067558

[B40] Pires S. F. Tolezano G. C. da Costa S. S. Kawahira R. S. H. Kim C. A. Rosenberg C. (2020). Expanding the role of SETD5 haploinsufficiency in neurodevelopment and neuroblastoma. Pediatr. Blood Cancer 67, e28376. 10.1002/pbc.28376 32748512

[B41] Powis Z. Farwell Hagman K. D. Mroske C. McWalter K. Cohen J. S. Colombo R. (2018). Expansion and further delineation of the SETD5 phenotype leading to global developmental delay, variable dysmorphic features, and reduced penetrance. Clin. Genet. 93, 752–761. 10.1111/cge.13132 28881385

[B42] Rauch A. Wieczorek D. Graf E. Wieland T. Endele S. Schwarzmayr T. (2012). Range of genetic mutations associated with severe non-syndromic sporadic intellectual disability: An exome sequencing study. Lancet 380, 1674–1682. 10.1016/S0140-6736(12)61480-9 23020937

[B43] Rincon-Arano H. Halow J. Delrow J. J. Parkhurst S. M. Groudine M. (2012). UpSET recruits HDAC complexes and restricts chromatin accessibility and acetylation at promoter regions. Cell. 151, 1214–1228. 10.1016/j.cell.2012.11.009 23177352 PMC3518625

[B44] Scattoni M. L. Crawley J. Ricceri L. (2009). Ultrasonic vocalizations: A tool for behavioural phenotyping of mouse models of neurodevelopmental disorders. Neurosci. Biobehav. Rev. 33, 508–515. 10.1016/j.neubiorev.2008.08.003 18771687 PMC2688771

[B45] Sessa A. Fagnocchi L. Mastrototaro G. Massimino L. Zaghi A. Indrigo M. (2019). SETD5 regulates chromatin methylation state and preserves global transcriptional fidelity during brain development and neuronal wiring. Neuron 104, 271–289. e13. 10.1016/j.neuron.2019.07.013 31515109

[B46] Seto E. Yoshida M. (2014). Erasers of histone acetylation: The histone deacetylase enzymes. Cold Spring Harb. Perspect. Biol. 6, a018713. 10.1101/cshperspect.a018713 24691964 PMC3970420

[B47] Shuib S. McMullan D. Rattenberry E. Barber R. M. Rahman F. Zatyka M. (2009). Microarray based analysis of 3p25-p26 deletions (3p- syndrome). Am. J. Med. Genet. A 149, 2099–2105. 10.1002/ajmg.a.32824 19760623

[B48] Skarnes W. C. Rosen B. West A. P. Koutsourakis M. Bushell W. Iyer V. (2011). A conditional knockout resource for the genome-wide study of mouse gene function. Nature 474, 337–342. 10.1038/nature10163 21677750 PMC3572410

[B49] Stur E. Soares L. A. Louro I. D. (2017). SETD5 gene variant associated with mild intellectual disability – A case report. Genet. Mol. Res. 16, 1–6. 10.4238/gmr16029615 28549204

[B50] Szczałuba K. Brzezinska M. Kot J. Rydzanicz M. Walczak A. Stawiński P. (2016). SETD5 loss-of-function mutation as a likely cause of a familial syndromic intellectual disability with variable phenotypic expression. Am. J. Med. Genet. A 170, 2322–2327. 10.1002/ajmg.a.37832 27375234

[B51] Vuong H. E. Yano J. M. Fung T. C. Hsiao E. Y. (2017). The microbiome and host behavior. Annu. Rev. Neurosci. 40, 21–49. 10.1146/annurev-neuro-072116-031347 28301775 PMC6661159

[B52] Wang Z. Hausmann S. Lyu R. Li T.-M. Lofgren S. H. Flores N. M. (2020). SETD5-coordinated chromatin reprogramming regulates adaptive resistance to targeted pancreatic cancer therapy. Cancer Cell. 37, 834–849. e13. 10.1016/j.ccell.2020.04.014 32442403 PMC8187079

[B53] White J. K. Gerdin A.-K. Karp N. A. Ryder E. Buljam M. Bussell J. N. (2013). Genome-wide generation and systematic phenotyping of knockout mice reveals new roles for many genes. Cell. 154, 452–464. 10.1016/j.cell.2013.06.022 23870131 PMC3717207

[B54] Yang Z. Zhang C. Che N. Feng Y. Li C. Xuan Y. (2021). Su(var)3-9, enhancer of Zeste, and Trithorax domain-containing 5 facilitates tumor growth and pulmonary metastasis through up-regulation of AKT1 signaling in breast cancer. Am. J. Pathol. 191, 180–193. 10.1016/j.ajpath.2020.10.005 33129761

[B55] Yang Z. Zhang C. Liu X. Chen N. Feng Y. Xuan Y. (2022). SETD5 regulates glycolysis in breast cancer stem-like cells and fuels tumor growth. Am. J. Pathol. 192, 712–721. 10.1016/j.ajpath.2021.12.006 35063407

[B56] Yu H. Sun J. Zhao C. Wang H. Liu Y. Xiong J. (2019). SET domain containing protein 5 (SETD5) enhances tumor cell invasion and is associated with a poor prognosis in non-small cell lung cancer patients. BMC Cancer 19, 736. 10.1186/s12885-019-5944-2 31345185 PMC6659235

[B57] Yu S. E. Kim M. S. Park S. H. Yoo B. C. Kim K. H. Jang Y. K. (2017). SET domain-containing protein 5 is required for expression of primordial germ cell specification-associated genes in murine embryonic stem cells. Cell. biochem. Funct. 35, 247–253. 10.1002/cbf.3269 28612505

[B58] Zhang A. Yeung P. L. Li C.-W. Tsai S.-C. Dinh G. K. Wu X. (2004). Identification of a novel family of ankyrin repeats containing cofactors for p160 nuclear receptor coactivators. J. Biol. Chem. 279, 33799–33805. 10.1074/jbc.M403997200 15184363

[B59] Zhang L. Ma H. (2012). Complex evolutionary history and diverse domain organization of SET proteins suggest divergent regulatory interactions. New Phytol. 195, 248–263. 10.1111/j.1469-8137.2012.04143.x 22510098

[B60] Zhang Y. Yan L. Yao W. Chen K. Xu H. Ye Z. (2019). Integrated analysis of genetic abnormalities of the histone lysine methyltransferases in prostate cancer. Med. Sci. Monit. 25, 193–239. 10.12659/MSM.912294 30616239 PMC6330996

