# Supplementary material for: Neurobehavioral characteristics of mice with SETD5 mutations as models of IDD23 and KBG syndromes
Source: Front Genet. 2023 Jan 4;13:1022339. doi: 10.3389/fgene.2022.1022339 (PMC9846138; doi:10.3389/fgene.2022.1022339)
Supplement: Supplementary file 2 [file Image1.PDF]

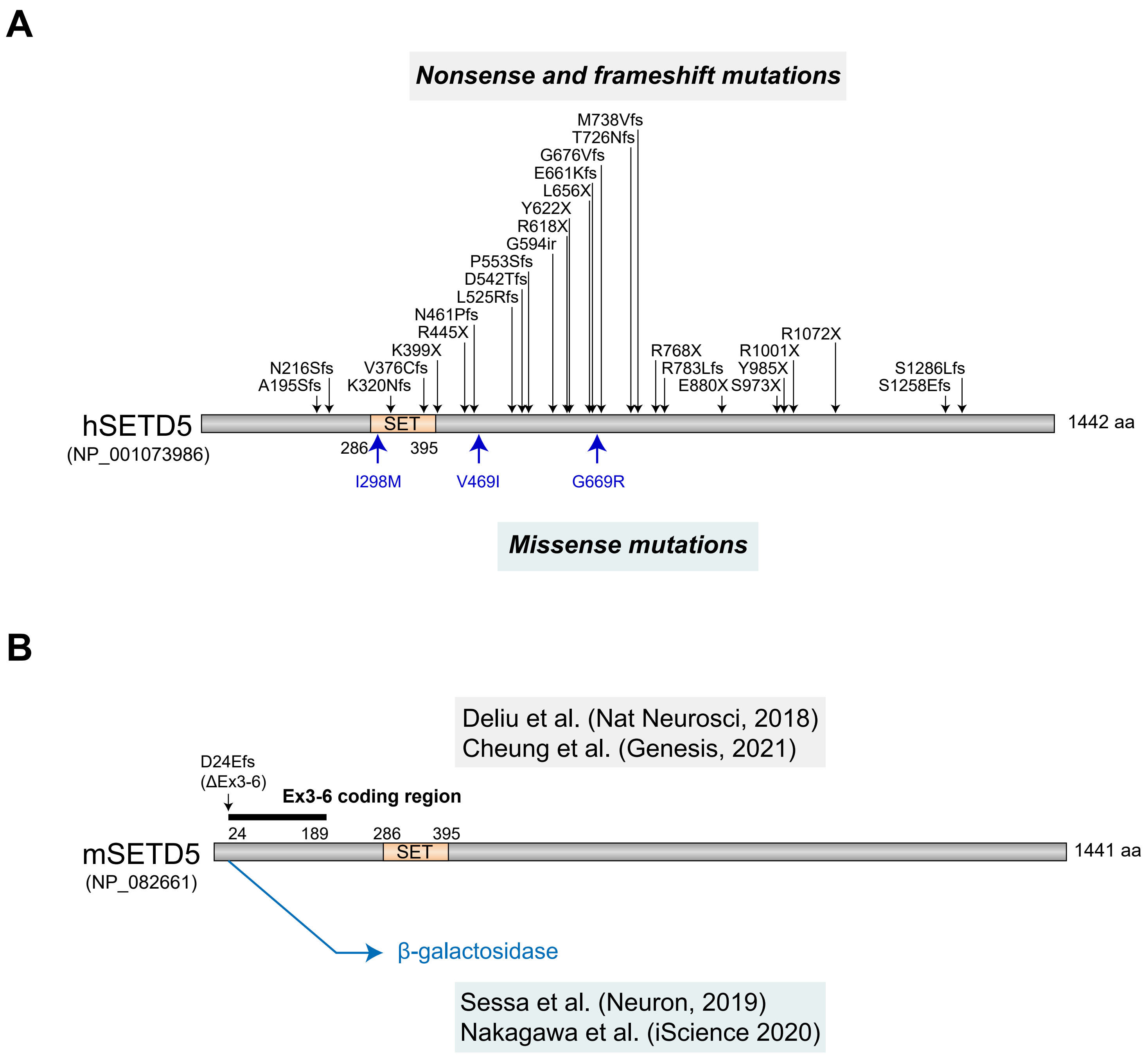

Supplementary Figure: Domain structure of human and mouse SETD5 with the locations of mutations. (**A**) Domain structure of human SETD5. Nonsense, frameshift and missense mutations found in IDD and KBG syndrome patients are shown. Individual amino acid is represented by signle letter notation. SET, Su(var)3-9, Enhancer-of-zeste and Trithorax; X, stop codon; fs, frame-shift; ir; intron retention. (**B**) Domain structure of mouse SETD5. *Setd5* mutant mice in Deliu et al. (Nat Neurosci, 2018) and Cheung et al. (Genesis, 2021) harbor mutation resulting in D to E substitution at 24th amino acid redisue followed by frameshit, while Sessa et al. (Neuron, 2019) and Nakagawa et al. (iScience, 2020) utilized mice which contain β-galactosidase fused to first 23 amino acid residues in place of wild-type SETD5. *Setd5* mutant mice in Moore et al. (Transl Psychiatry, 2019) have *GFP-polyA* cassette located at the upstream of the translation initiation sequence of *Setd5*, resulting in the expression of GFP where wild-type SETD5 is otherwise expressed (not shown).
